# Evaluating the Effectiveness of Medical–Dental Integration to Close Preventive and Disease Management Care Gaps

**DOI:** 10.3389/fdmed.2021.670012

**Published:** 2021-07-02

**Authors:** David M. Mosen, Matthew P. Banegas, John F. Dickerson, Jeffrey L. Fellows, Daniel J. Pihlstrom, Hala M. Kershah, Jason L. Scott, Erin M. Keast

**Affiliations:** 1 Center for Health Research, Kaiser Permanente Northwest, Portland, OR, United States,; 2 Permanente Dental Associates, Portland, OR, United States,; 3 Dental Administration, Kaiser Permanente Northwest, Portland, OR, United States

**Keywords:** integration, Medicare, chronic conditions, preventive, elderly health

## Abstract

**Background:**

The integration of medical care into the dental setting has been shown to facilitate the closure of care gaps among patients with unmet needs. However, little is known about whether program effectiveness varies depending on whether the care gap is related to preventive care or disease management.

**Materials and Methods:**

We used a matched cohort study design to compare closure of care gaps between patients aged 65+ who received care at a Kaiser Permanente Northwest (KPNW) Medical–Dental Integration (MDI) clinic or a non-MDI dental clinic between June 1, 2018, and December 31, 2019. The KPNW MDI program focuses on closing 12 preventive (e.g., flu vaccines) and 11 disease management care gaps (e.g., HbA1c testing) within the dental setting. Using the multivariable logistic regression, we separately analyzed care gap closure rates (yes vs. no) for patients who were overdue for: (1) preventive services only (*n* = 1,611), (2) disease management services only (*n* = 538), or (3) both types of services (*n* = 429), analyzing closure of each care gap type separately. All data were obtained through the electronic health record of KPNW.

**Results:**

The MDI patients had significantly higher odds of closing preventive care gaps (OR = 1.51, 95% CI = 1.30–1.75) and disease management care gaps (OR = 1.65, 95% CI = 1.27–2.15) than the non-MDI patients when they only had care gaps of one type or the other. However, no significant association was found between MDI and care gap closure when patients were overdue for both care gap types.

**Conclusions:**

Patients with care gaps related to either preventive care or disease management who received dental care in an MDI clinic had higher odds of closing these care gaps, but we found no evidence that MDI was helpful for those with both types of care gaps.

**Practical Implications:**

MDI may be an effective model for facilitating the delivery of preventive and disease management services, mainly when patients are overdue for one type of these services. Future research should examine the impact of MDI on long-term health outcomes.

## INTRODUCTION

Poor oral health, particularly periodontal disease is associated with many common systemic health conditions, including diabetes (1–7), cardiovascular disease (CVD) (7, 8), coronary artery disease (CAD) (9–11), cerebral vascular disease (9, 11–13), hypertension (HTN) (14, 15), cancer (16) and rheumatoid arthritis (RA) (17, 18). Adults aged 65 years and older represent a population at high risk for both and oral and systemic disease, with over 65% having periodontal disease (19, 20) and over 80% having two or more chronic systemic diseases (21, 22).

Previous research has demonstrated that the dental setting can be an ideal setting to promote preventive health for those at risk of developing chronic systemic conditions. For example, Jontell and Glick (23) found that dental healthcare providers can effectively screen and identify patients for serious complications from CVD that they may not be aware of. A systematic review (24) found that screening for dysglycemia in dental clinics was effective in identifying individuals who required triage for further glycemic management.

In a recently published study, we found that a comprehensive medical–dental integration (MDI) program at the Kaiser Permanente Northwest (KPNW) was successful at facilitating the closure of medical care gaps among a population of patients aged 65 years and older who were overdue for either preventive (e.g., flu vaccines) or disease management medical services (e.g., HbA1c testing for persons with diabetes) (25). This research was the first to show that the dental setting can facilitate the provision of recommended evidence-based preventive and disease management medical services for elderly populations (26–28).

Although we found that MDI at KPNW was effective at closing care gaps for preventive and disease management care gaps, there is limited research on whether the effectiveness of MDI on care gaps closure differs by service type: preventive medicine or disease management. For example, MDI is more effective at closing preventive medicine services, such as flu vaccines as opposed to disease management services, such as HbA1c testing for adults with diabetes. Accordingly, the primary objective of this study was to separately examine the association of MDI with the closure of medical care gaps among populations overdue for (1) preventive and/or (2) disease management care gaps. The rationale for conducting this stratified analysis was for program evaluation purposes, namely to understand whether care gaps were closed differently based on the type of medical care gap. Such information can be used for quality improvement purposes to revise and refine the MDI programs.

## MATERIALS AND METHODS

The methods for this analysis were presented previously (25). The study population included a retrospective matched cohort analysis of KPNW medical and dental members (*n* = 2,578) who received care at any of four MDI clinics of KPNW and those who received care at a non-MDI clinic during the same time period (*n* = 2,578; total *n* = 5,156). Briefly, we identified all patients who met four inclusion criteria: (1) aged 65 or older; (2) had a dental visit at a KPNW MDI dental clinic between June 1, 2018 and December 31, 2019; (3) had at least one medical care gap at the time of their first (index) dental visit during that time period; and (4) had 12 months of continuous health plan enrollment prior to index dental visit. Using a matching algorithm, we then identified a 1:1 matched sample of patients who met the inclusion criteria except that their index dental visit occurred at one of 13 non-MDI dental clinics during the same time period. Patients from each MDI dental clinic were matched with patients from one of three to four non-MDI dental clinics with a similar total volume of dental staff full-time equivalents (FTEs) and an annual volume of dental visits. Patients were also matched based on sex, care gap type (preventive only, disease management only, or both), age (within 5 years), and index visit date (±60 days). MDI and non-MDI were further propensity-matched based on seven characteristics: Charlson comorbidity index (CCI; 0, 1, 2+); smoking status (yes vs. no); emergency department (ED) utilization in the previous 12 months (any vs. none); hospitalization in previous 12 months (any vs. none); the presence of any of five systemic conditions [diabetes mellitus (DM), RA, CVD, CAD, and HTN; yes vs. no]; periodontal disease status (healthy/early, moderate, and advanced); and a total number of open care gaps at the index visit (continuous).

To assess whether care gap closure varied by service type, we stratified the population into three cohorts based on care gap type(s) at baseline: (1) preventive care gaps only, (2) disease management care gaps only, and (3) both preventive and disease management care gaps. A list of all care gaps and their categories has been described previously (25). Data from the electronic health record (EHR) of KPNW were used for matching and analysis.

### The IRB Approval

The protocol for this study was approved by the institutional review board (IRB) within KPNW, who approved a waiver of individual consent for data use (IRB# 00000405 and FWA# 00002344). As this was a “data-only” no-patient contact study, all data storage and analytic practices adhered to the research compliance standards of KPNW.

### Setting

The KPNW is a comprehensive healthcare system that currently serves ~605,000 medical members and 250,000 dental members in Oregon and Washington. The KPNW MDI program includes three distinct model types employed in four dental clinics that are in their level of integration (25). The MDI model with the least integration was implemented on June 1st, 2018; the other two models were implemented on August 1st, 2018. Each model is described below.

#### Least Integration

This model consists of a dental office located in the same building as medical offices (e.g., lab, vision, and nurse treatment center for immunizations) with no medical staff embedded in the dental office. In this model, a dental member assistant (DMA) identifies care gaps at the time of dental visits using the EHR and coordinates closely with other medical departments located within the building to complete the overdue care gaps.

#### Moderate Integration

In this model, a licensed practice nurse (LPN) is embedded within a stand-alone dental clinic to address care gaps. The LPN can provide immunizations, collect samples for lab-based tests, and provide other basic services [e.g., HbA1c testing, BP screening, and DM foot exams] directly in the dental setting. The LPN also coordinates all other medical services that require offsite referrals (e.g., mammography) or offsite follow-up with primary care (e.g., follow-up regarding abnormal HbA1c results).

#### Most Integration

This model, implemented in two KPNW locations, consists of co-located medical and dental offices with an embedded LPN within the dental clinic itself. The LPN provides direct services and coordinates with other co-located medical staff to complete additional services. At both clinics following this model, a DMA works closely with the LPN to identify care gaps prior to dental visits. The LPN then provides service to close care gaps that can be directly addressed in the dental setting (e.g., immunizations) and coordinates with other co-located medical departments to address other care gaps after the dental visit (e.g., DM retinopathy screening). The LPN also arranges follow-up care as needed with primary care for care coordination regarding chronic conditions.

##### Non-MDI dental offices

Non-MDI dental offices have no embedded medical staff to complete on-site care gap closure or care coordination to complete needed follow-up services. Within the non-MDI clinics, the dental staff uses an EHR-based decision support tool (described below) to remind patients of any care gaps they may have.

### Identification of Medical Care Gaps and Outcome Measures

The KPNW dental and medical clinics use an EHR-based program called the panel support tool (PST) to identify patient care gaps. The PST, which has been in use since 2006 (29), uses informatics to track care gaps, patient reminders, and follow-up care (29). The PST lists care gaps based on the current clinical guidelines and evidence for ongoing screening tests and disease management services (26–28).

The primary outcome measure of this study was closure of *all* relevant (1) preventive and (2) disease management medical care gaps present at the index dental visit in each of the three care gap cohorts. For 18 of the 23 measures, care gap closure was assessed at 30 days following the index visit; fecal immunochemical testing, mammography, annual DM exam, retinopathy exam, and smoking cessation were assessed at 60 days following the index visit.

### Statistical Analysis

As part of the stratified analysis, we first conducted descriptive analyses of analytic variables to confirm assumptions and as a quality assurance process. In order to assess the performance of our matching algorithm, we calculated standardized differences of demographic and clinical characteristics between MDI and non-MDI patients within each of the three care gap cohorts (Tables 1.1–1.3). We used a threshold of ≥0.2 to identify variables that are meaningfully different between groups (30); this threshold has been used previously in observational studies (31, 32). Because no differences of 0.2 or higher were found after matching, we did not conduct further adjustment in the stratified regression analysis. Finally, we used three separate logistic regression models to compare rates of closure of care gaps between MDI and non-MDI patients within each of the three care gap cohorts (Table 2).

## RESULTS

### Population Characteristics: Care Gap Cohorts

The preventive care gap cohort included 62.5% (*N* = 3,222) of the study population, whereas the disease management cohort included 20.9% (*N* = 1,076) and the combined preventive and disease management gap cohort included 16.6% (*N* = 858) of the population. Within the three cohorts, patients in the MDI and non-MDI groups were well balanced for care gap type, age, and sex, area deprivation index (ADI) (33) (as a proxy for socioeconomic status), and most clinical and demographic variables after matching (Tables 1.1–1.3). Although there was variation between the three cohorts with respect to demographics, comorbidities, systemic conditions, periodontal disease status and open care gaps at baseline, none of these differences exceeded the standard difference ≥0.2.

### Association Between MDI and Care Gap Closure by Cohort

Table 2 shows the results of the logistic regression analyses testing associations between MDI and care gap closure for each cohort. In the preventive care gap and disease management cohorts, patients seen at MDI clinics had 1.51 the odds of closing all preventive gaps and 1.65 the odds of closing all disease management gaps than patients seen at non-MDI clinics. In the cohort of patients with both types of care gaps, there was no significant association being seen at an MDI clinic and closure of all care gaps.

## DISCUSSION

This study found that among patients with only preventive or disease management care gaps, receiving care in MDI clinics was associated with significantly greater odds of care gap closure than receiving care at non-MDI clinics. However, there was no significant difference in rates of closure of all care gaps between MDI and non-MDI patients among those with both preventive and disease management care gaps at their index visit. Taken with previously published results by our research team that found the KPNW MDI program was effective at closing care gaps overall (when combining all three care gap cohorts) (25), these findings suggest that the success of the KPNW MDI program may be driven by those who do not have both preventive and disease management care gaps—these cohorts made up over 80% of the eligible population.

This is the first study of which we are aware to examine the association of MDI with the closure of specific types of care gaps. Our findings suggest that MDI is effective at facilitating the use of needed medical services for either preventive or disease management care, suggesting the broad benefits of this approach. This research is consistent with research demonstrating the success of other integration efforts in the dental setting. A study by Jontell and Glick (23) found that oral healthcare professionals can successfully screen and identify patients who are not aware of their risk of developing serious complications from CVD and advise these individuals to seek medical care. Similarly, a recent study (34) found that screening for dysglycemia in dental clinics were effective at identifying high-risk patients who required triage for glycemic management.

The findings from our research have clear program significance. Currently, Medicare does not pay for dental services, except if the care is related to hospitalization. Because of the clear benefit in promoting the use of preventive and disease management services among the Medicare population, our results suggest that there may be benefits in the Medicare program offering dental insurance coverage to recipients aged 65 years and over.

We recognize several limitations associated with this analysis. First, all data were collected in one healthcare system, potentially limiting generalizability to other populations. While the KPNW membership reflects the underlying population of the area (29, 35), it has a higher proportion older than age 65 compared to the US population overall, suggesting that more research may be needed to determine if these strategies are equally effective in other populations specifically those who are younger (36). Another limitation is that the retrospective cohort design we used does not allow us to assess causality: other differences between MDI and non-MDI clinics, and patients could account for some of the differences between groups. However, this limitation is reduced due to a robust propensity matching of the samples, which reduced the potential impact of confounders on results.

## CONCLUSION AND FUTURE RESEARCH

For about 80% of patients in our study, visiting an MDI clinic was associated with higher odds of closing all care gaps than visiting a non-MDI clinic. For patients overdue for both preventive and disease management of care gaps, we found no significant association between MDI and care gap closure.

Further research is needed to understand why there was no difference in care gap closure between MDI and non-MDI for those with both types of care gaps upon dental visit. Possible factors include more gaps to close at baseline and potentially less adherence among patients with both types of care gaps. In addition, future research should directly study the costs and benefits of the MDI model. For health systems and policymakers to evaluate the broader implementation of MDI, it is critical to understand the financial costs and benefits of closing medical-related care gaps in the dental setting, especially for the Medicare population. These could include savings due to improved long-term health outcomes of patients whose care gaps are closed due to MDI.

## Figures and Tables

**TABLE 1.1 |:** Population Characteristics: Preventive Care Gaps Only.

Population characteristics	MDI Clinics *N* = 1,611	Non-MDI Clinics *N* = 1,611	*p*-value	Standardized Difference
**Baseline gap type**				
Preventive alone	1,611 (100.0%)	1,611 (100.0%)	NA	NA
**Exact-matched variables**				
Age (mean ± S.D.; matched within 5 yrs.)	70.6 ± 5.0	70.8 ± 5.1	0.18	0.05
**Sex (exact match)**				
Male (vs. female)	580 (36.0%)	580 (36.0%)	1.00	0.00
**Propensity-matched variables**			0.04	
CCI 0	1,010 (62.7%)	946 (58.7%)		0.04
CCI 1	237 (14.7%)	282 (17.5%)		0.05
CCI 2+	364 (22.6%)	383 (23.8%)		0.02
**Current smoker**				
Yes (vs. no)	2 (0.1%)	6 (0.4%)	0.16	0.04
**ED use in previous 12 months**				
Yes (vs. no)	247 (15.3%)	276 (17.1%)	0.17	0.03
**Hospitalization in previous 12 months**				
Yes (vs. no)	99 (6.1%)	130 (8.1%)	0.03	0.05
**Systemic conditions (% yes)**				
Diabetes-Mellitus (DM)	166 (10.3%)	179 (11.1%)	0.46	0.02
Rheumatoid Arthritis (RA)	27 (1.7%)	26 (1.6%)	0.89	0.00
Cardiovascular Disease (CVD)	147 (9.1%)	181 (11.2%)	0.05	0.05
Cardiovascular Disease (CAD)	143 (8.9%)	179 (11.1%)	0.03	0.05
Hypertension (HTN)	624 (38.7%)	644 (40.0%)	0.47	0.02
Periodontal disease status			0.87	
Healthy/early	1,273 (79.0%)	1,276 (79.2%)		0.00
Moderate	236 (14.7%)	227 (14.1%)		0.01
Advanced	37 (2.3%)	35 (2.2%)		0.01
Missing	65 (4.0%)	73 (4.5%)		0.02
Total open gaps at index visit (mean ± S.D.)	1.6 ± 0.9	1.6 ± 0.9	0.94	0.00
**Non-Propensity matched variables**				
SES^b^: Area Deprivation Index (ADI) (Mean ± SD)	4.4 ± 2.4	4.5 ± 2.6	0.33	0.04

*N* = 3,222 (62.5% of total population)^a^.

a Population includes population of Medicare patients age ≥ 65 with 1+ care gaps at baseline. *P*-value from t-test for age, count of open gaps at baseline, and ADI state rank; *P*-value from chi-square for all other variables.

b SES, socioeconomic status.

**TABLE 1.2 |:** Population Characteristics: Disease Management Gaps Only.

Population characteristics	MDI Clinics *N* = 538	Non-MDI Clinics *N* = 538	*p*-value	Standardized Difference
**Baseline gap type**				
Disease management alone	538 (100.0%)	538 (100.0%)	NA	NA
**Exact-matched variables**				
Age (mean ± S.D.; matched within 5 yrs.)	72.5 ± 5.4	72.6 ± 5.4	0.77	0.02
Sex (exact match)				
Male (vs. female)	285 (53.0%)	285 (53.0%)	1.00	0.00
**Propensity-matched variables**			0.73	
CCI 0	154 (28.6%)	149 (27.7%)		0.01
CCI 1	122 (22.7%)	133 (24.7%)		0.03
CCI 2+	262 (48.7%)	256 (47.6%)		0.01
**Current smoker**				
Yes (vs. no)	82 (15.2%)	73 (13.6%)	0.44	0.03
**ED use in previous 12 months**				
Yes (vs. no)	93 (17.3%)	96 (17.8%)	0.81	0.01
**Hospitalization in previous 12 months**				
Yes (vs. no)	44 (8.2%)	46 (8.6%)	0.83	0.01
**Systemic conditions (% yes)**				
Diabetes-Mellitus (DM)	325 (60.4%)	320 (59.5%)	0.76	0.01
Rheumatoid Arthritis (RA)	5 (0.9%)	7 (1.3%)	0.56	0.03
Cardiovascular Disease (CVD)	79 (14.7%)	75 (13.9%)	0.73	0.01
Cardiovascular Disease (CAD)	106 (19.7%)	95 (17.7%)	0.39	0.04
Hypertension (HTN)	429 (79.7%)	419 (77.9%)	0.46	0.02
Periodontal disease status			0.32	
Healthy/early	355 (66.0%)	372 (69.1%)		0.03
Moderate	124 (23.1%)	123 (22.9%)		0.00
Advanced	27 (5.0%)	23 (4.3%)		0.02
Missing	32 (6.0%)	20 (3.7%)		0.07
Total open gaps at index visit (mean ± S.D.)	1.5 ± 0.9	1.4 ± 0.9	0.47	0.04
Non-Propensity matched variables				
SES^b^: Area Deprivation Index (ADI) (Mean ± SD)	4.9 ± 2.2	4.5 ± 2.4	0.01	0.16

*N* = 1,076 (20.9% of total population)^a^.

a Population includes population of Medicare patients age ≥ 65 with 1+ care gaps at baseline. *P*-value from t-test for age, count of open gaps at baseline, and ADI state rank; *P*-value from chi-square for all other variables.

b SES, socioeconomic status.

**TABLE 1.3 |:** Population Characteristics: Both Gaps.

Population characteristics	MDI Clinics *N* = 429	Non-MDI Clinics *N* = 429	*p*-value	Standardized Difference
**Baseline gap type**				
Both (Preventive and Management)	429 (100.0%)	429 (100.0%)	NA	NA
**Exact-matched variables**				
Age (mean ± S.D.; matched within 5 yrs.)	69.8 ± 3.9	70.1 ± 4.1	0.34	0.06
**Sex (exact match)**				
Male (vs. female)	175 (40.8%)	175 (40.8%)	1.00	0.00
**Propensity-matched variables**			0.55	
CCI 0	187 (43.6%)	173 (40.3%)		0.04
CCI 1	91 (21.2%)	102 (23.8%)		0.04
CCI 2+	151 (35.2%)	154 (35.9%)		0.01
**Current smoker**				
Yes (vs. no)	74 (17.2%)	72 (16.8%)	0.86	0.01
**ED use in previous 12 months**				
Yes (vs. no)	58 (13.5%)	62 (14.5%)	0.69	0.02
**Hospitalization in previous 12 months**				
Yes (vs. no)	23 (5.4%)	26 (6.1%)	0.66	0.02
**Systemic conditions (% yes)**				
Diabetes-Mellitus (DM)	216 (50.3%)	220 (51.3%)	0.79	0.01
Rheumatoid Arthritis (RA)	5 (1.2%)	4 (0.9%)	0.74	0.02
Cardiovascular Disease (CVD)	37 (8.6)	48 (11.2%)	0.21	0.06
Cardiovascular Disease (CAD)	60 (14.0%)	53 (12.4%)	0.48	0.03
Hypertension (HTN)	312 (72.7%)	322 (75.1%)	0.44	0.02
Periodontal disease status			0.53	
Healthy/early	289 (67.4%)	288 (67.1%)		0.00
Moderate	91 (21.2%)	80 (18.7%)		0.04
Advanced	18 (4.2%)	20 (4.7%)		0.02
Missing	31 (7.2%)	41 (9.6%)		0.06
Total open gaps at index visit (mean ± S.D.)	3.3 ± 1.4	3.4 ± 1.6	0.42	0.06
**Non-propensity matched variables**				
SES^b^: Area Deprivation Index (ADI) (Mean ± SD)	5.1 ± 2.5	4.8 ± 2.7	0.20	0.09

*N* = 858 (16.6% of total population)^a^.

a Population includes Medicare patients age ≥ 65 with 1+ care gaps at baseline. *P*-value from t-test for age, count of open gaps at baseline, and ADI state rank; *P*-value from chi-square for all other variables.

b SES, socioeconomic status.

MDI clinics = dental offices with integrated medical and dental services.

**TABLE 2 |:** Logistic Regression Analysis of Medical Care Gap Closure^a^, by Care Gap Cohort.

	OR	95 % CI	p-value
**Population with Preventive Care Gaps Only (*N* = 3,222)**			
MDI Population	1.51	1.30–1.75	<0.0001
Non-MDI Population	1.00	–	–
**Population with Disease Management Care Gaps Only (*N* = 1,076)**			
MDI Population	1.65	1.27–2.15	0.0002
Non-MDI Population	1.00	—	—
**Population with both Preventive and Disease Management Care Gaps (*N* = 858)**			
MDI Population	0.94	0.62–1.41	NS
Non-MDI Population	1.00	—	—

a Population includes Medicare patients age ≥65 with 1+ care gaps at baseline. All care gap closure assessed at 30 days post index visit, except for FIT testing, mammography, annual DM exam, retinopathy exam, and smoking cessation which was assessed at 60 days post index visit. *p*-value from Logistic Regression Analysis. NS, not statistically significant at *p* value < 0.05.

## Data Availability

The datasets presented in this article are not readily available because this data is proprietary information held by Kaiser Permanente Northwest. Questions regarding data access should be directed to david.m.mosen@kpchr.org.
